# The serine proteinase hepsin is an activator of pro-matrix metalloproteinases: molecular mechanisms and implications for extracellular matrix turnover

**DOI:** 10.1038/s41598-017-17028-3

**Published:** 2017-12-01

**Authors:** David J. Wilkinson, Antoine Desilets, Hua Lin, Sarah Charlton, Maria del Carmen Arques, Adrian Falconer, Craig Bullock, Yu-Chen Hsu, Kristian Birchall, Alastair Hawkins, Paul Thompson, William R. Ferrell, John Lockhart, Robin Plevin, Yadan Zhang, Emma Blain, Shu-Wha Lin, Richard Leduc, Jennifer M. Milner, Andrew D. Rowan

**Affiliations:** 1 Skeletal Research Group, Institute of Genetic Medicine, Newcastle University, Central Parkway, Newcastle upon Tyne, NE1 3BZ UK; 2 Department of Pharmacology-Physiology, Faculty of Medicine and Health Sciences, Institut de Pharmacologie, Université de Sherbrooke, Sherbrooke, Québec, J1H 5N4 Canada; 3 Department of Clinical Laboratory Sciences and Medical Biotechnology, National Taiwan University, Taipei, Taiwan; 4 LifeArc, Stevenage Bioscience Catalyst, Gunnels Wood Road, Stevenage, SG1 2FX UK; 5 Institute for Cell and Molecular Biosciences, Newcastle University, Newcastle upon Tyne, UK; 6 Institute of Infection, Immunity and Inflammation, University of Glasgow, Glasgow, UK; 7 Institute of Biomedical and Environmental Health Research, University of the West of Scotland, Paisley, UK; 8 Strathclyde Institute of Pharmacy and Biomedical Sciences, University of Strathclyde, Glasgow, UK; 9 Arthritis Research UK Biomechanics and Bioengineering Centre, Cardiff University, Cardiff, UK; 10 Arthritis Research UK, Copeman House, St Mary’s Gate, Chesterfield, Derbyshire S41 7TD UK

## Abstract

Increasing evidence implicates serine proteinases in the proteolytic cascades leading to the pathological destruction of extracellular matrices such as cartilage in osteoarthritis (OA). We have previously demonstrated that the type II transmembrane serine proteinase (TTSP) matriptase acts as a novel initiator of cartilage destruction via the induction and activation of matrix metalloproteinases (MMPs). Hepsin is another TTSP expressed in OA cartilage such that we hypothesized this proteinase may also contribute to matrix turnover. Herein, we demonstrate that addition of hepsin to OA cartilage in explant culture induced significant collagen and aggrecan release and activated proMMP-1 and proMMP-3. Furthermore, hepsin directly cleaved the aggrecan core protein at a novel cleavage site within the interglobular domain. Hepsin expression correlated with synovitis as well as tumour necrosis factor α expression, and was induced in cartilage by a pro-inflammatory stimulus. However, a major difference compared to matriptase was that hepsin demonstrated markedly reduced capacity to activate proteinase-activated receptor-2. Overall, our data suggest that hepsin, like matriptase, induces potent destruction of the extracellular matrix whilst displaying distinct efficiencies for the cleavage of specific substrates.

## Introduction

Aberrant proteolysis is a characteristic of pathological tissue remodelling events including osteoarthritis (OA), the most common of arthritic diseases. OA is a progressively degenerative disease causing severe pain, disability and morbidity. A key characteristic of OA is the breakdown of the cartilage extracellular matrix (ECM), a tissue that provides smooth surfaces to allow joint articulation. The chondrocyte is the only cell-type responsible for ECM homeostasis as well as the production of proteinases involved in both health as well as disease. Aggrecan provides water retention and compressive strength via its hydrophilic properties whilst type II collagen provides structural integrity. A subset of the matrix metalloproteinases (MMPs), the collagenases (predominantly MMP-1 and MMP-13), specifically degrade native type II collagen^1^ which is considered an irreversible step in disease^2^. Despite this understanding, MMP inhibition has not proven therapeutically beneficial to prevent cartilage matrix destruction, due to a lack of inhibitor specificity and a range of off-target effects^1^.

ECM breakdown often occurs in the pericellular space such as for the chondrocyte in OA cartilage^3^, making membrane-associated proteinases ideal candidates for an initiating role in tissue turnover. Serine proteinases are also key enzymes that contribute to the proteolytic destruction of the cartilage ECM by activating latent proMMPs which is a key rate-limiting step (see^4^ and references therein). We have previously shown that the type II transmembrane serine proteinase (TTSP) matriptase is expressed in OA cartilage, and can induce and activate proMMP-1 and -3^5^. The destruction of cartilage collagen and aggrecan by matriptase is dependent upon metalloproteinase activities and the activation of proteinase-activated receptor-2 (PAR2)^5,6^. We have previously reported roles for PAR2 in inflammatory joint disease^7^ and OA^8,9^ and shown PAR2 to be important in the production of pro-inflammatory mediators known to drive ECM breakdown such as interleukin-(IL-)1 and tumour necrosis factor α (TNFα)^10^, two cytokines which also upregulate PAR2 expression in cartilage^11^.

In general, TTSPs have diverse substrate specificities^12^ although matriptase exhibits many similarities to the related TTSP, hepsin. Their overlapping substrate repertoires have been widely reported^13–16^, with hepsin being most highly expressed in liver but also in many other adult tissues^17^. Like matriptase, hepsin is implicated in a number of diseases associated with pathological ECM remodeling, including cancer (reviewed in^16,18^). Since we have also detected hepsin expression in human OA cartilage^5^, in this study we assessed the impact hepsin activity has on ECM destruction using cartilage and OA as a disease model of ECM turnover.

## Results

### Hepsin induces *ex vivo* ECM degradation

**Figure 1:**
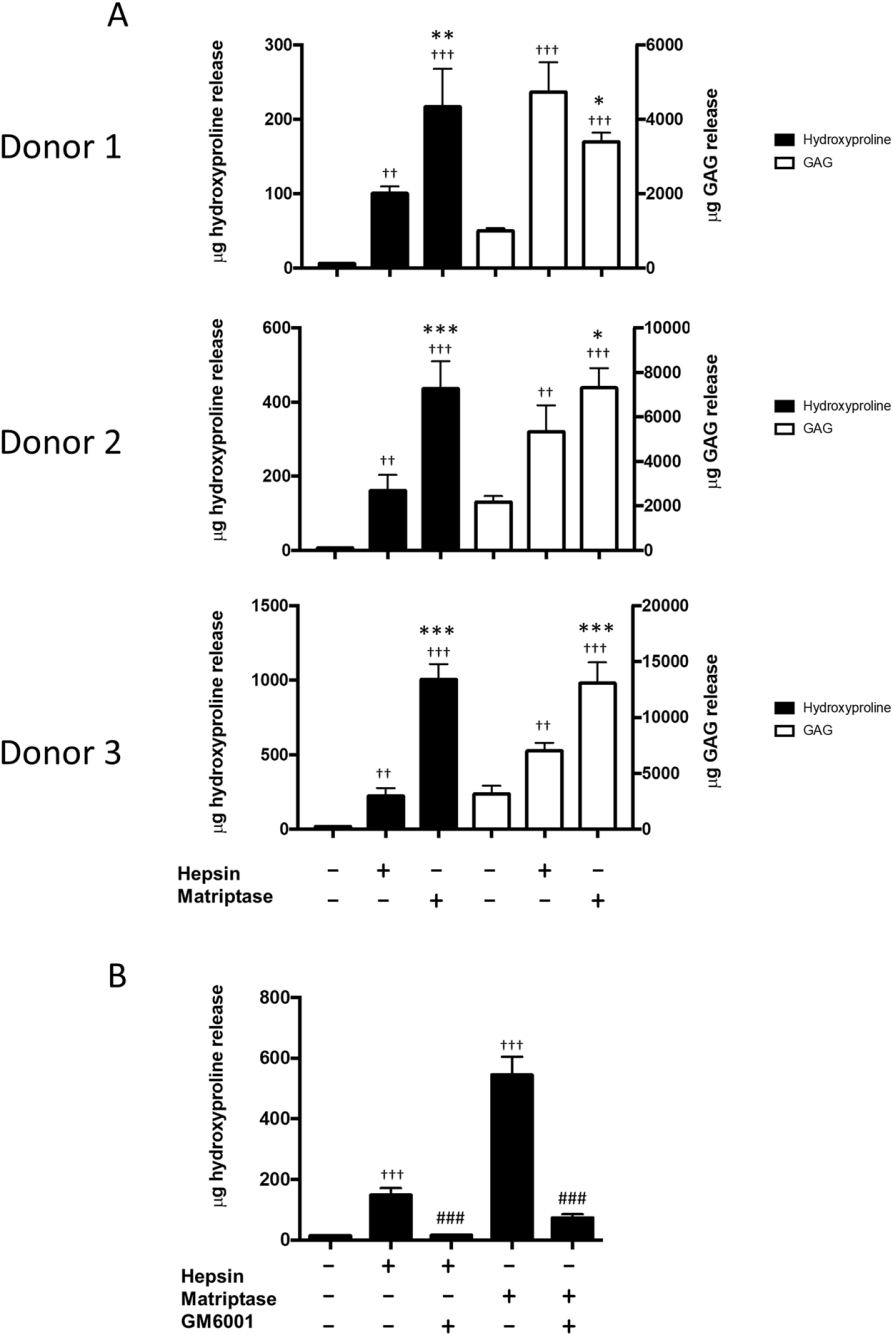
Hepsin induces cartilage degradation in OA cartilage explant cultures. (**A**) Hepsin or matriptase (100 nM) was added to human OA knee cartilage in explant culture. Cartilage from donor #1 (85 y, M) and #2 (77 y, F) were incubated for 3 days whilst donor #3 (66 y, F) was incubated for 7 days. (**B**) Hepsin or matriptase were added to human OA cartilage in the presence or absence of the metalloproteinase inhibitor GM6001 (10 µM). Collagen and aggrecan release were measured by hydroxyproline and DMMB assays, respectively. Statistical analyses were performed using one-way ANOVA with Bonferroni post-hoc test where: *p < 0.05, ***p < 0.001 versus hepsin; and ^††^p < 0.01, ^†††^p < 0.001 versus control; ^###^p < 0.001 versus hepsin or matriptase only. Data are plotted as mean ± SD and representative of at least 3 independent experiments (n = 4).

To test whether hepsin was capable of inducing cartilage matrix destruction, recombinant enzyme was incubated with human OA cartilage explant cultures. Hepsin reproducibly induced collagen release from this tissue (Fig. 1A) although this was consistently significantly lower than that induced by equimolar amounts of matriptase. Hepsin also induced potent aggrecan degradation but the levels of release, compared to matriptase, demonstrated variability between patient samples. As seen previously for matriptase^5,6^, inclusion of the broad-spectrum metalloproteinase inhibitor, GM6001, reproducibly blocked the observed hepsin-induced collagen release (Fig. 1B).

### Hepsin is a proMMP activator

**Figure 2:**
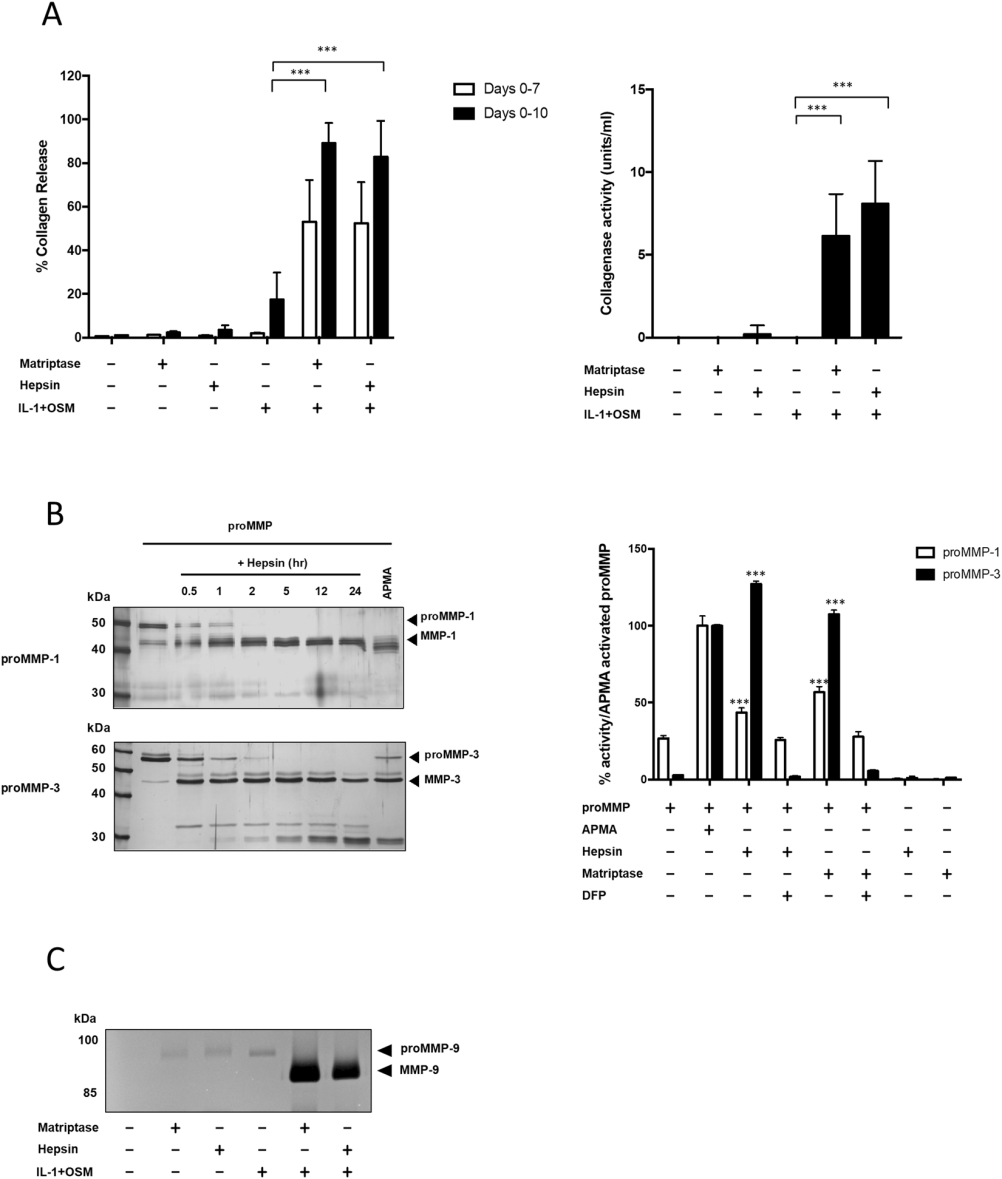
Hepsin is an activator of proMMP-1 and proMMP-3. (**A**) Bovine nasal cartilage explants were stimulated with IL-1 (1 ng/ml) and OSM (10 ng/ml) in the presence and absence of matriptase or hepsin (100 nM). Media were removed and the cartilage re-stimulated at day 7. The experiment was terminated as described in the experimental procedures, and the percentage collagen release determined by hydroxyproline assay. *Left panel:* Data are presented as day 7 collagen release and total collagen release (day 7 + 10). *Right panel:* Collagenase activity in Day 7 media was monitored by a ^3^H collagen bioassay. Data are plotted as mean ± SD (n = 6) and representative of 3 independent experiments. Statistical significance was measured by one-way ANOVA with Bonferroni post-hoc test where ***p < 0.001 vs IL-1+OSM. (**B**) Recombinant full-length proMMP-1 or proMMP-3 were incubated with recombinant hepsin in a 1:5 serine proteinase:MMP molar ratio at 37 °C for up to 24 h. *Left panel:* Products were separated by 10% SDS-PAGE and gels stained with silver. *Right panel:* For activity assays, proMMP-1 or proMMP-3 were incubated with hepsin or matriptase for 4 h at 37 °C. Enzyme to substrate ratios were 1:5 for proMMP-1 and 1:10 for proMMP-3. MMP activity was assessed with the MMP-specific substrate FS-6. ProMMP alone or in the presence of APMA were used as negative and positive controls, respectively. Hepsin and matriptase without MMP did not cleave the substrate significantly. Hepsin and matriptase were also pre-treated with the irreversible serine proteinase inhibitor di-isopropyl fluorophosphate (DFP), to demonstrate the involvement of serine proteinase activity in MMP activation. Data are presented as a percentage of APMA-activated proMMP (mean ± SD) following subtraction of the blank measurement. Statistical significance was measured by a one-way ANOVA with Bonferroni post-hoc test where ***p < 0.001 compared to proMMP alone. (**C**) Day 7 culture media from stimulated bovine cartilage in A were subjected to gelatin zymography. Where gel images have been cropped for clarity, full length images are presented in supplementary information.

To assess whether hepsin can activate proMMPs we used cytokine-stimulated bovine cartilage explant cultures. In this *ex vivo* model of cartilage breakdown, addition of proMMP activators induces early collagen release^5,19^. As seen previously, low levels of collagen release were observed at day 7 following stimulation with IL-1+OSM^5^. The addition of hepsin led to a significant increase in collagenolysis which was observed at both day 7 and 10 of culture (Fig. 2A, left panel). These data correlated with the levels of active collagenase detected in the media of these cultures which were similar for both hepsin- and matriptase-treated cartilages (Fig. 2A, right panel). Hepsin processed proMMP-1 and -3 into proteins corresponding to the molecular masses of the mature enzymes (approximately 43 kDa and 46 kDa, respectively; Fig. 2B, left panel), whilst MMP activity assays confirmed this processing generated active MMP-1 and MMP-3 (Fig. 2B, right panel). Although gelatin zymography indicated proMMP-9 activation following hepsin addition (Fig. 2C), this was most probably due to proMMP-3 activation since MMP activity assays failed to demonstrate hepsin-mediated activation of proMMP-9 as well as proMMP-8 and -13 (Supplementary Figure 1).

### Hepsin is an aggrecan-degrading enzyme

**Figure 3:**
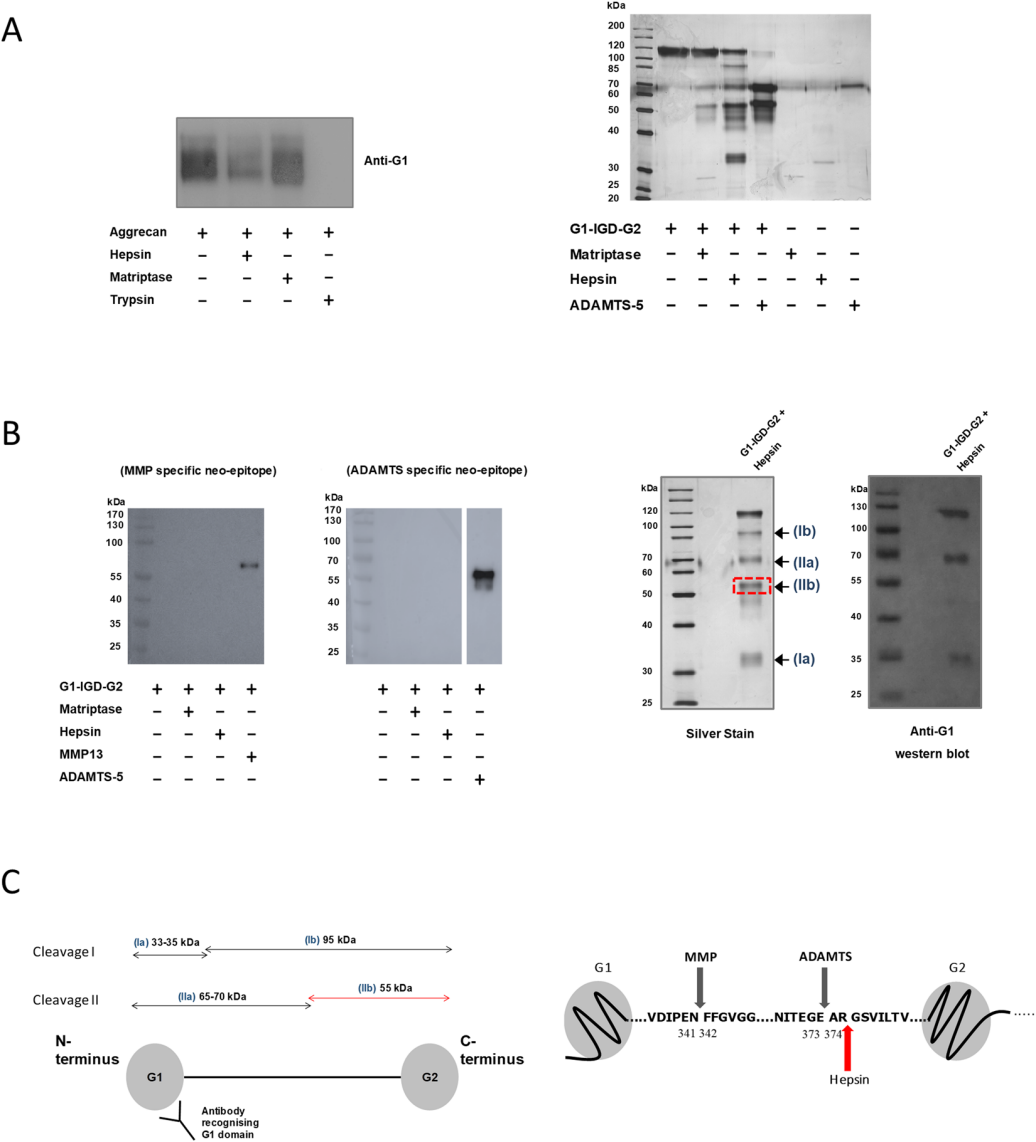
Hepsin degrades aggrecan. (**A**) Hepsin cleaves within the interglobular domain of aggrecan. *Left panel:* Deglycosylated bovine aggrecan (400 nM) was incubated with hepsin, matriptase or trypsin (all 10 nM) for 16 hours at 37 °C. Incubation products were separated on a 7.5% SDS-PAGE gel and western blotted using an antibody recognising the aggrecan G1 domain. Results are representative of 3 independent experiments. *Right panel*: Hepsin generates a novel cleavage within the aggrecan IGD. Recombinant human G1-IGD-G2 was incubated with matriptase or hepsin in a 1:20 enzyme to substrate ratio (156 nM G1-IGD-G2 and 7.8 nM proteinase) for 16 h at 37 °C then resolved by SDS-PAGE and stained with silver. (**B**) *Left panel:* In a similar experiment, products were western blotted using BC-3 and BC-14 neoepitope antibodies. Incubation of G1-IGD-G2 with ADAMTS-5 (1.6 μg/ml) and MMP-13 (6 μg/ml) acted as positive controls for aggrecanase and MMP cleavage, respectively. *Right panel:* G1-IGD-G2 (156 nM) was incubated with hepsin (7.8 nM) for 8 h at 37 °C. Products (*Ia, Ib, IIa* and *IIb*) were separated by SDS-PAGE and stained with silver, western blotted using an anti-G1 aggrecan antibody or blotted onto PVDF and stained with Coomassie brilliant blue R250, after which the desired band (*IIb*) was excised and subjected to N-terminal sequencing. Where images have been cropped for clarity, full-length gels/blots are presented in supplementary information. The bands on each figure are taken from the same gels/blots under the same exposure conditions. (**C**) N-terminal sequencing identifies novel cleavage site. *Left panel:* Schematic representation of the hepsin-cleaved aggrecan fragments based on data from **B**. The desired product (*IIb*; red line), corresponding to the new N-terminus following cleavage (red dashed box in **B**), was excised from a Coomassie-stained blot. *Right panel*: Known MMP and aggrecanase cleavage sites within the aggrecan IGD including the novel hepsin cleavage at Arg^375^↓Ser^376^ confirmed by N-terminal sequencing (highlighted by red arrow).

The variable level of aggrecan release induced by hepsin (Fig. 1A) led us to assess whether hepsin was capable of direct aggrecan cleavage. Interestingly, hepsin caused proteolysis of the aggrecan core protein, as demonstrated by anti-G1 immunoblotting (Fig. 3A, left panel). To determine whether hepsin cleaved within the pathologically-relevant aggrecan interglobular domain (IGD), incubation with recombinant G1-IGD-G2 (corresponding to amino acids Val^20^-Gly^675^ of the aggrecan core protein) confirmed cleavage within this region (Fig. 3A, right panel) at sites distinct from known metalloproteinase cleavage sites (Fig. 3B, left panel). Matriptase also processed this protein, albeit with markedly weaker efficiency. N-terminal sequencing of the hepsin-mediated cleavage product (Fig. 3B, right panel) indicated a novel cleavage site at Arg^375^↓Gly^376^ in close proximity to the known aggrecanase cleavage site (Fig. 3C).

### Hepsin expression correlates with synovial inflammation

**Figure 4:**
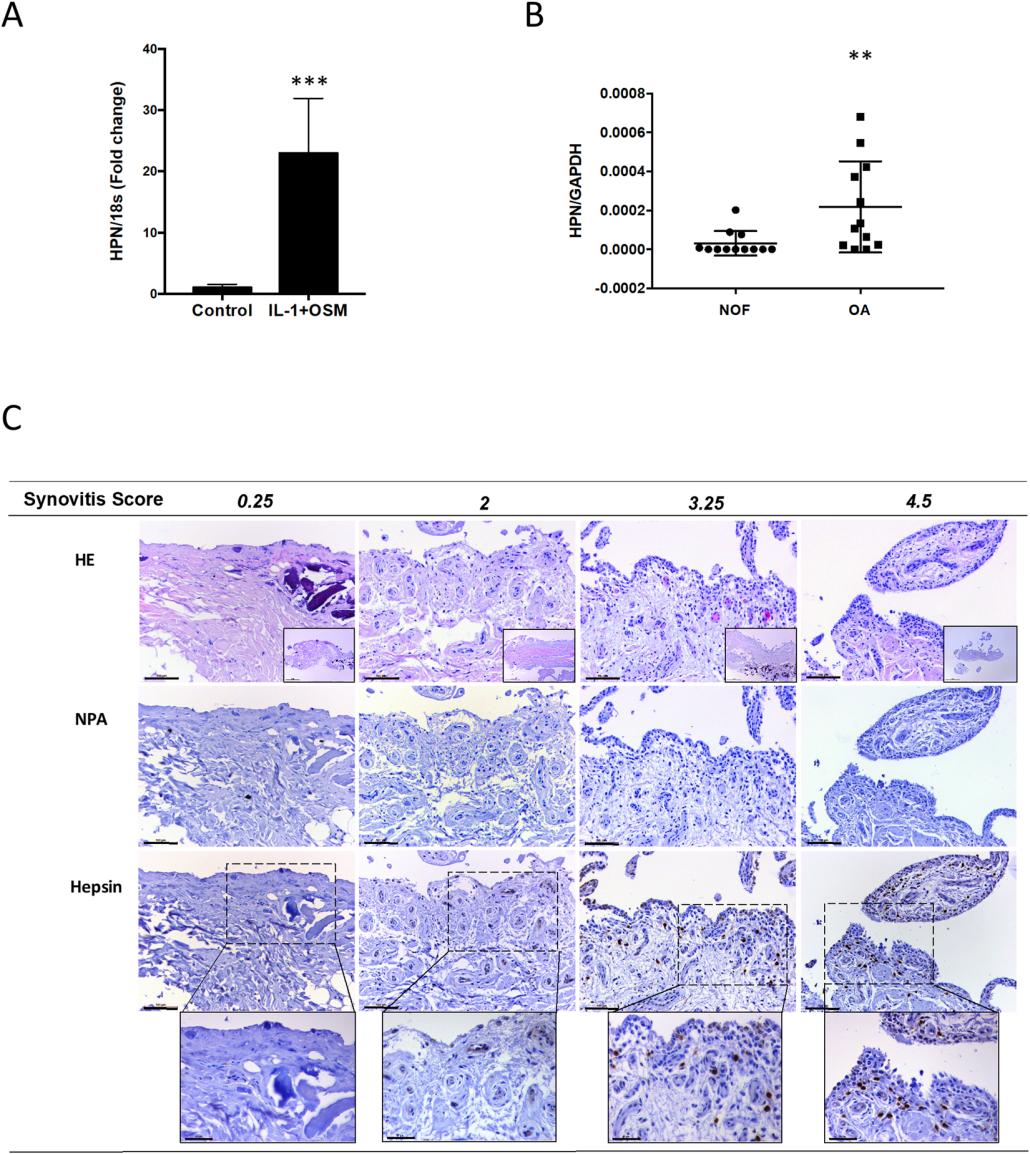
Hepsin expression is associated with inflammation. (**A**) Bovine articular cartilage was excised from metacarpophalangeal joints and stimulated with IL-1 (5 ng/ml) and OSM (10 ng/ml) for 4 days. RNA was isolated and reverse transcribed prior to real-time PCR. Data were normalized to the level of *18 S* rRNA and plotted as mean ± SD fold change (n = 4). Significant differences were determined using a t-test where ***p < 0.001. (**B**) Gene expression levels in hip synovium from patients with OA (closed circles; n = 12) or normal controls (NOF) (open squares; n = 12) of *HPN* were determined as described in the Experimental procedures and normalized to the level of glyceraldehyde 3-phosphate dehydrogenase (GAPDH). HPN expression was undetected in 8/12 NOF samples and 2/12 OA samples, and significant differences between the normal and OA groups were determined using a two-sided Mann-Whitney U test, where **p < 0.01. (**C**) Human OA synovia samples were fixed, embedded in paraffin, sectioned and stained with haematoxylin and eosin. Synovitis was scored blinded by two independent scorers based on levels of inflammation, stromal activation and hyperplasia. Summed scores were totaled and described as no synovitis (0–1), slight synovitis (2–3), and moderate synovitis (4–6). Representative samples from within this scoring scale are presented where hepsin was detected by immunohistochemistry using a rabbit anti-hepsin polyclonal antibody. A control consisting of no primary antibody (NPA) was also performed. The scale bars represent 100 µm (except 50 µm for the insets).

Many proteinase genes are induced by pro-inflammatory mediators, including various cytokine combinations such as IL-1+OSM, in chondrocytes and many other cell-types^20,21^. In this study, we found this cytokine combination also markedly induced hepsin expression (Fig. 4A) in cartilage but, interestingly, not matriptase (Supplementary Figure 2). Since inflammation is recognized to be a contributing factor in OA, especially TNFα (reviewed in^22^), we assessed hepsin expression in OA synovia and found it to be significantly elevated compared to normal (neck of femur; NOF) synovial tissues (Fig. 4B). Notably, there appeared to be two distinct groups in the OA samples: one with very little hepsin expression as per the normal group, whilst the second had markedly higher expression. Interestingly, overall hepsin expression correlated with TNFα expression levels (r = 0.57, p = 0.0034; Supplementary Figure 3). Furthermore, immunohistochemistry confirmed the presence of hepsin in OA synovial samples, which correlated with histological grade of synovitis (Fig. 4C).

### Hepsin is a weak PAR2 activator

**Figure 5:**
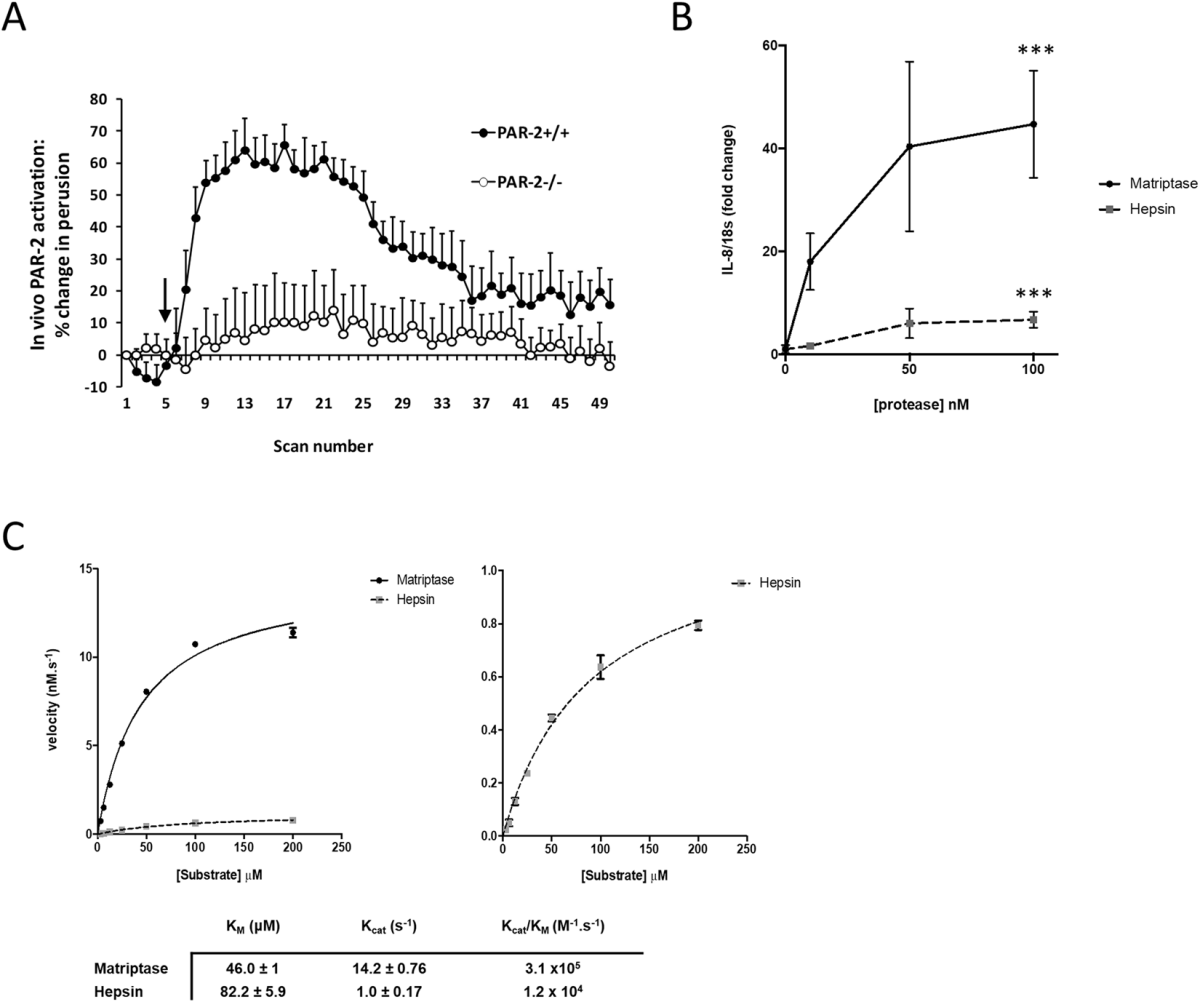
Hepsin is a weak PAR2 activator. (**A**) To assess the ability of hepsin to activate PAR2 *in vivo*, hepsin (5 µg) was administered topically to an exposed knee joint of both PAR2^+/+^ (●; n = 3) and PAR2^−/−^ (○; n = 4) mice, with synovial perfusion monitored by high resolution Doppler imaging, as described previously^5^. Data are presented as percentage change in perfusion from the baseline mean ± SEM. (**B**) Hepsin or matriptase (10–100 nM) were added to SW1353 cells stably expressing PAR2 for 3 hours. Cells were lysed, mRNA reverse transcribed and *IL8* expression monitored by real-time PCR. Data were normalized to an *18S* rRNA housekeeping gene and plotted as fold change compared to unstimulated cells. (**C**) Matriptase is a more potent PAR2 activator. *Left panel:* Hepsin (1 nM) or matriptase (1 nM) were incubated with 2-Abz-SKGRSLIG-Y(NO_2_) (0–200 μM), fluorescence monitored and linear reaction velocities obtained from progress curves. Velocities were plotted against substrate concentration and Michaelis-Menten constants generated by non-linear regression. *Right panel:* Expanded view of the non-linear regression for hepsin confirming normal Michaelis-Menten kinetics. Curves are representative of 3 independent experiments. Tabulated kinetic constants are also shown as mean ± SD across each of 3 independent experiments.

**Figure 6:**
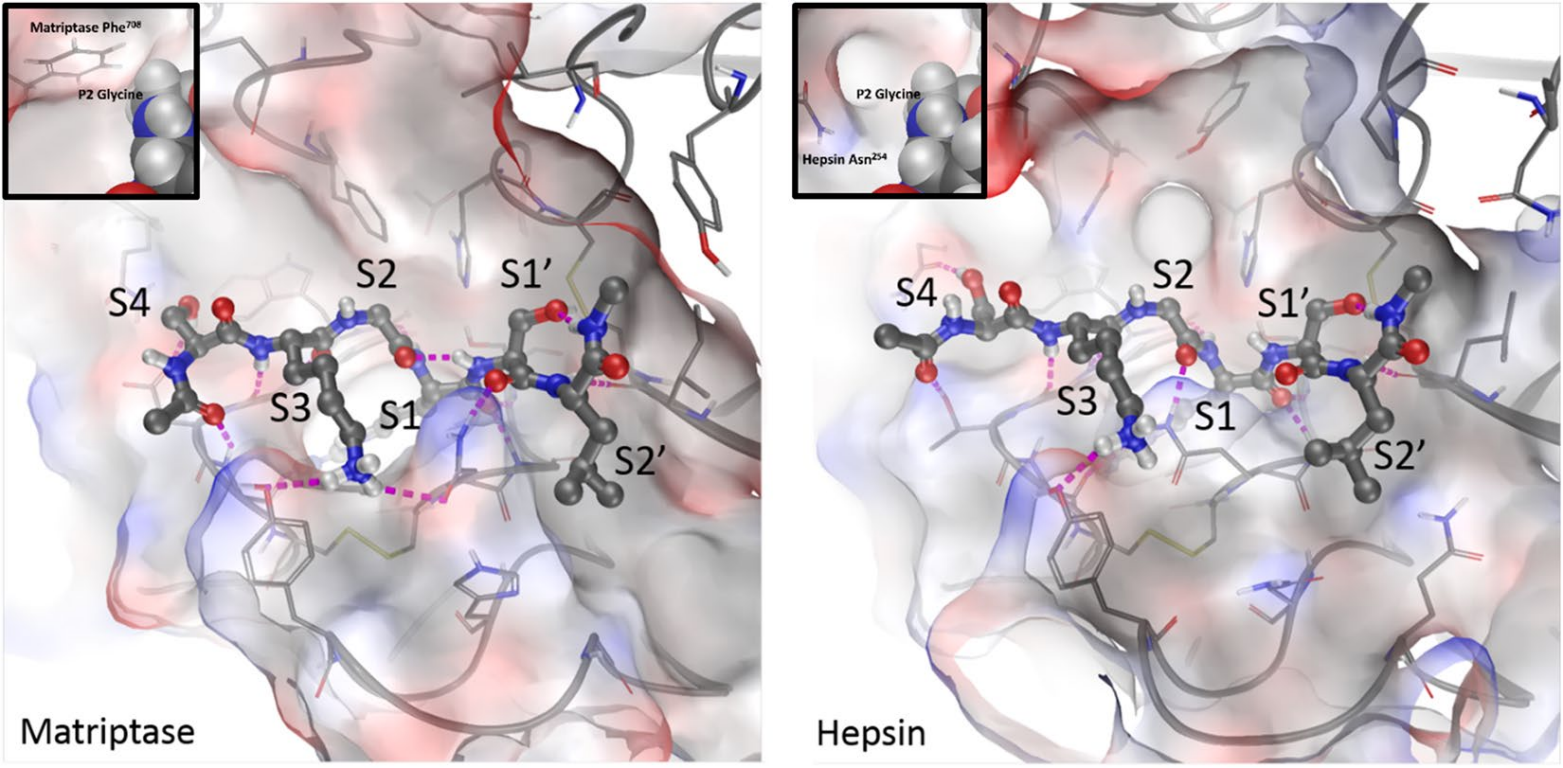
Structural modelling of hepsin explains poor PAR2 activation. The PAR2 cleavage sequence peptide (SKGR↓SL) is docked into matriptase and hepsin and displayed side-by-side for comparison. The peptide is shown in ball and stick representation, receptor surfaces are coloured by electrostatic potential, H-bonding interactions are indicated with pink dashes and sub-pockets labelled. The binding sites are largely similar and the peptide binds in the same way. The most significant difference is in the S2′ pocket where the deep hydrophobic channel in matriptase better accommodates the Leu sidechain of the peptide compared to the polar shallow pocket in hepsin. The S2 pocket is also a significant point of difference with the peptide Gly residue engaging in Van der Waals contacts with the Phe sidechain in matriptase, which is absent in the more open pocket of hepsin (insets show a close up of the S2 pocket from a different angle with the peptide in a spacefill format, including hydrogen atoms).

Since we have previously demonstrated that matriptase directly induced MMP expression from cartilage via PAR2 activation^5^, we investigated the ability of hepsin to activate PAR2. Following topical administration of hepsin to murine joints, as reported previously for matriptase^5^, hepsin induced a rapid and reproducible increase in synovial perfusion in wild type PAR2^+/+^ mice compared to PAR2^−/−^ mice (Fig. 5A). However, although the perfusion assay confirmed hepsin can activate PAR2, this assay requires non-physiological amounts of enzyme. Therefore, we next quantified receptor activation using SW1353 chondrocytes transfected to constitutively express PAR2. Addition of hepsin to these cells reproducibly induced the expression of the PAR2-dependent gene *IL8*; however, an equimolar amount of matriptase stimulated significantly more *IL8* expression (approx. 6-7 times at 50–100 nM) in the PAR2-expressing cells (Fig. 5B). Non-transfected SW1353 cells failed to respond (Supplementary Figure 4). To assess PAR2 activation kinetics, a quenched fluorescent peptide mimicking the PAR2 activation sequence confirmed hepsin cleaved with significantly reduced catalytic efficiency compared to matriptase (Fig. 5C, left panel) albeit with normal Michaelis-Menten kinetics (Fig. 5C, right panel). Finally, we assessed whether subsite specificity and structural differences within the active site architecture of matriptase and hepsin could explain the significantly different levels of PAR2 activation displayed by these proteinases. Substrate specificity comparisons^23^ highlighted a marked preference of matriptase for the amino acids in the P2 and P2′ positions of the PAR2 activation sequence (SKGR↓SLIG). To further explore these observations, we conducted molecular docking of the SKGRSL sequence in the active sites of both matriptase and hepsin which indicated that the bulky hydrophobic P2′ Leu sidechain is better accommodated in the deep hydrophobic channel of matriptase compared to the more shallow and polar S2′ pocket offered by hepsin. Furthermore, the Gly residue in the P2 position of the PAR2 peptide has better complementarity to the receptor surface in matriptase, engaging in Van der Waals contacts with a Phe residue that is absent from the more open S2 pocket of hepsin (Fig. 6). These structural data are consistent with the notion that hydrolysis of the PAR2 activation sequence (SKGR↓SLIG) by hepsin is considerably less favourable than that provided by matriptase.

## Discussion

Despite our understanding of the importance of metalloproteinases in ECM destruction, and the requirement for proMMP activation^24–26^, the interplay between serine and metalloproteinases is still an emerging paradigm^4^. Moreover, human OA cartilage is particularly resistant to resorption *in vitro*, and we have previously observed that although addition of potent cytokine stimuli such as IL-1 + OSM produce high proMMP levels, significant collagen release is rarely observed^6,20^. It is now believed that serine and metalloproteinase cascades are interconnected during cartilage catabolism, and such interplay likely functions in other connective tissue pathologies. The identity of specific serine proteinases, however, has remained elusive. Recent genomic and proteomic advances helped discover entirely novel families of proteinases, including TTSPs^16,18^. Of this family, we previously reported matriptase expression to be elevated in end-stage OA, identifying it to have a putative role as an initiator of cartilage collagen destruction by inducing the expression and, crucially, activation of proMMPs^5^. Herein, we now describe a second TTSP, hepsin, can also activate proMMPs and thus promote ECM turnover. Moreover, as shown previously for matriptase which induced early collagen release^5^, hepsin also increased collagenase activity to comparable levels suggesting similar capabilities in activating relevant proMMPs that promote collagenolysis. *In vitro* proMMP activation experiments further confirmed hepsin and matriptase display similar propensities for the activation of both proMMP-1 and proMMP-3. Indeed, hepsin activity is important for prostate and ovarian cancer cell invasion, a process linked to metalloproteinase activity in many circumstances^27–30^.

Data from an OA model now indicate that ECM remodeling events are phasic, with significant changes occurring throughout the disease course primarily as a result of changes to the joint microenvironment^31^; in reality, these genotypic/phenotypic fluctuations are likely in many pathological matrix remodelling scenarios. Moreover, it is now widely accepted that inflammation (typically cytokine-mediated) is a contributing factor that drives and perpetuates ECM turnover in OA^32,33^ as well as being a prime effector of matrix remodeling in many inflammatory pathologies. Our finding that hepsin is markedly up-regulated in chondrocytes in response to the catabolic stimulus IL-1 + OSM^20,21^ suggests hepsin expression is dynamic and highly regulated. Furthermore, this expression was consistent with the concomitant up-regulation of MMP substrates such as proMMP-1 and proMMP-3 to effect ECM turnover^21,34^.

The importance of proMMP-3 activators in the destruction of ECMs cannot be understated since this proteinase is capable of activating many other MMPs including MMP-13^35^. Although hepsin activation of proMMP-1 was relatively weak *in vitro*, proteolytic attack of proMMP-1 by a serine proteinase typically produces a partially active MMP which only becomes fully active following further cleavage by active MMP-3^36,37^. It is well established, for example, that cartilage ECM destruction involves multiple interdependent proteolytic cascades, the corollary of which is that some proteinases must function as upstream initiators. Interestingly, matriptase and hepsin undergo auto-activation, a unique phenomenon consistent with TTSPs functioning in this role^38^ although hepsin has also been reported to activate pro-matriptase^17^. With the initial stages of cartilage destruction localised to the pericellular space, somewhat analogously to an ‘invasive front’, membrane-anchored serine proteinases are ideally positioned for proMMP activation and the subsequent destruction of the surrounding pericellular matrix. To our knowledge, this is the first report demonstrating the ability of hepsin to activate proMMPs, and may have significant implications in settings where metalloproteinase-dependent matrix turnover is prevalent.

An intriguing finding was that the aggrecan IGD is a substrate for hepsin, cleavage occurring very close to the reported aggrecanase processing site. Our previously reported P1-P1′ specificity data for hepsin^39^ are consistent with the observed cleavage at Arg^375^↓Gly^376^. Although it is likely that the majority of catabolic events in cartilage are effected by metalloproteinases, our data suggest hepsin has the potential to generate an aggrecan peptide (Phe^342^-Arg^375^) very similar to the bioactive Phe^342^-Glu^373^ peptide (generated by combined MMP and aggrecanase activities) known to contribute to cartilage breakdown^40^ in the absence of aggrecanase activity. Thus, hepsin acting as a proMMP activator may further exacerbate ECM turnover in aggrecan-rich ECMs. It has also been shown to cleave the structurally ubiquitous basement membrane component laminin-332, as indeed have several MMPs, which helps maintain epithelial-mesenchymal cohesion in tissues exposed to external forces; laminin-332 cleavage may generate pro-migratory signals to cells (see^41^ and references therein). Combined with its ability to activate pro-hepatocyte growth factor^42^, hepsin is therefore capable of promoting invasion and metastasis.

Since we have previously demonstrated that matriptase-induced breakdown of OA cartilage is PAR2-dependent^5,6^ and that PAR2 antagonism is protective^8^, we investigated the propensity for hepsin to activate PAR2 in comparison to matriptase. We demonstrated that hepsin cleaved and activated PAR2 *in vivo*, albeit in a somewhat non-physiological setting. Kinetic assessment of PAR2 cleavage however highlighted a significantly reduced capability of hepsin to cleave PAR2 compared to matriptase. Consistent with these findings, hepsin-deficient mice subjected to destabilisation of the medial meniscus (DMM) surgery to induce OA were not protected from cartilage damage (Supplementary Figure 5). We have previously demonstrated the importance of the matriptase/PAR2 axis in the DMM model^6,8,9^ whereby PAR2 activation is a significant contributor to the observed cartilage damage. We hypothesise little or no activation of PAR2 by hepsin in cartilage explains the lack of protection in the DMM-treated hepsin-deficient mice and accounts for the lower levels of hepsin-induced collagen release from human OA cartilage explants compared to matriptase, whilst the similar efficiencies of MMP activation for both TTSPs further support this assumption. Indeed, weak activation of PAR2 by hepsin (relative to matriptase) has been reported previously in a study which indicated that hepsin-mediated PAR2 activation was primarily matriptase-dependent due to its activation by hepsin^17^.

Canonical PAR2 activation occurs at..SKGR↓SLIG.. and experimental substrate preference data^23,39^ indicate that matriptase prefers small residues at P2 (Gly/Ala) whilst hepsin is more tolerant and generally prefers larger side chains (Ala/Gln/Pro/Leu). Thus, although Gly at this position in the PAR2 sequence is accepted by hepsin, it is a much more preferred requirement in matriptase. Moreover, structural modelling provides some rationale for the observed substrate difference; Asn^254^ in the S2 pocket of hepsin provides much more space but less favourable surface complementarity than the corresponding Phe^708^ in matriptase. The other key difference in substrate preference is at the P2′ position where the Leu residue in the PAR2 sequence is well tolerated in matriptase but much less so in hepsin.

**Figure 7:**
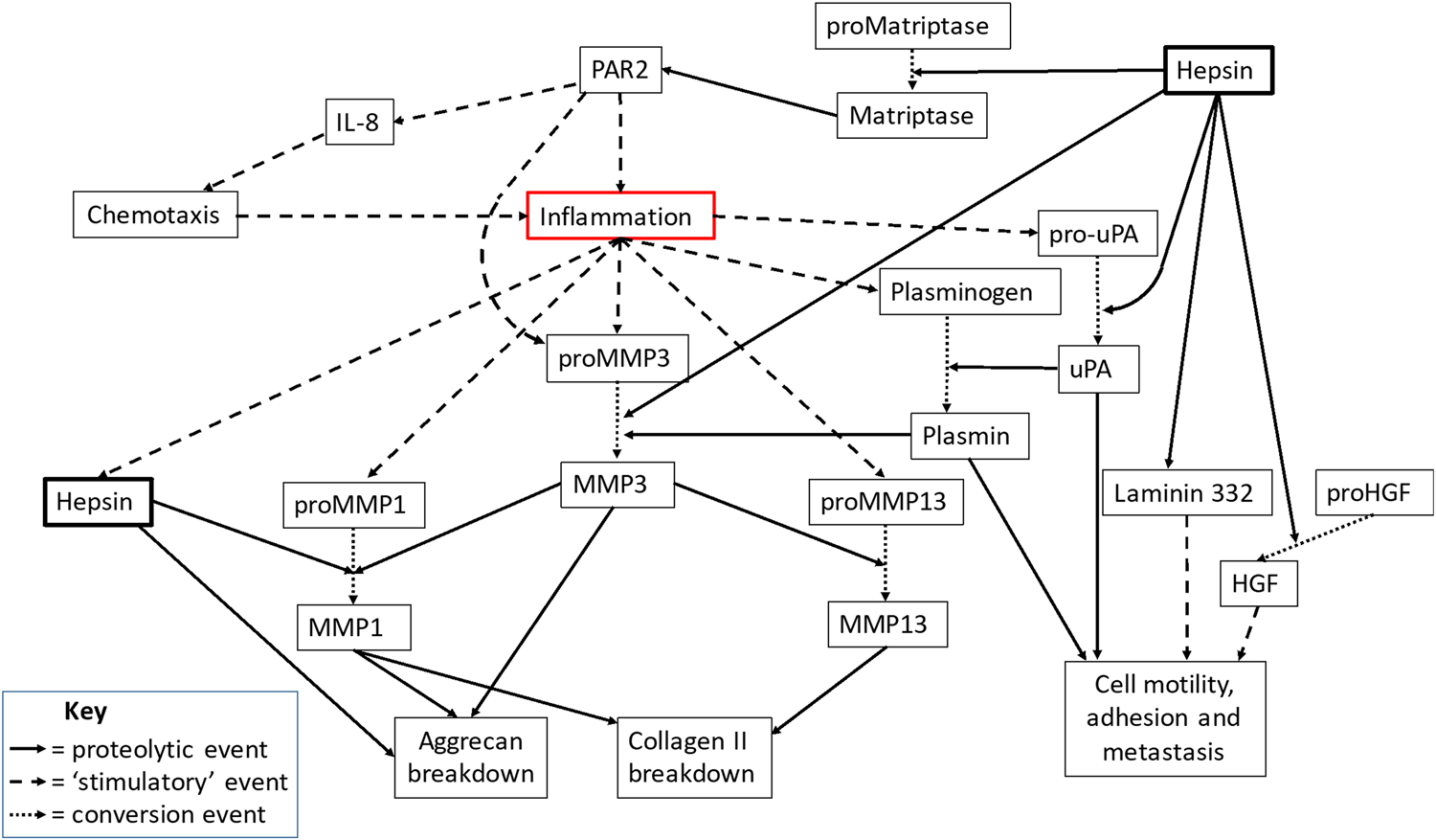
Schematic of the direct and indirect contributions of hepsin to alter ECM integrity. Firstly, hepsin activity is known to modulate cell motility via laminin 332^52^. Hepsin activates the uPA/plasminogen/plasmin cascade and also processes latent hepatocyte growth factor (HGF) leading to HGF/cMet signaling; together, these promote metastasis and altered epithelial cohesion^30,53^. Moreover, hepsin can activate pro-matriptase^17^ which leads to PAR2 activation and subsequent expression of proMMP-3 and proMMP-1^5^ as well as inflammatory mediators including IL-8^54^ leading to chemotaxis of inflammatory cells such as neutrophils and mast cells. Plasmin^55^, as well as hepsin, generate active MMP-3 which is a prime activator of many proMMPs including proMMP-1 and proMMP-13, key collagen-degrading proteinases. Inflammation induces the expression of prohepsin which auto-activates^17,38^ and can directly activate proMMP-1 as well as cleave within the aggrecan IGD to promote both aggrecanolysis and collagenolysis to further impair the structural integrity of tissues.

As hepsin gene expression is responsive to pro-inflammatory cytokines, and correlates with synovitis from human OA patients, we conclude that hepsin may have a role in cartilage destruction that has a more inflammatory phenotype. Indeed, increasing evidence supports a role for inflammation in OA progression amongst a subset of patients^33^ which mirrored our findings, whilst the DMM model is known to have little inflammatory involvement^43^. Thus, herein we demonstrate that hepsin is a proteinase that directly and indirectly promotes pathological ECM alterations (see Fig. 7). Indeed, we propose that its ability to function as an activator of proMMPs and other proteinase zymogens may have implications in not only arthritis but many other matrix remodeling scenarios such as cancer metastasis and invasion.

## Experimental procedures

### Reagents and antibodies

All chemicals of the highest purity available were obtained from Sigma Chemical Co (Poole, UK) unless otherwise stated. All cytokines and proteins used were recombinant human unless stated otherwise. IL-1α was a generous gift from Dr Keith Ray (GlaxoSmithKline, Stevenage, UK). Oncostatin M (OSM), proMMP-1, proMMP-13, matriptase (aa596-895) and hepsin (aa45-417) were produced in-house (see^6,12^ and references therein). Purified serine proteinases were active site titrated with the burst titrant 4-methylumbelliferyl-p-guanidinobenzoate. ProMMP-3 and proMMP-8 were purchased from R&D Systems (Abingdon, UK). GM6001 was purchased from Calbiochem (Nottingham, UK). The anti-G1 antibody was kindly provided by Dr John Mort (McGill University, Canada). BC-3 and BC-14 aggrecan antibodies were provided by Prof. Bruce Caterson (Cardiff University, UK). Ab25 was kindly provided by Dr Daniel Kirchhofer (Genentech, CA, USA) and produced as described^44^. The MMP substrate, fluorescence substrate-6 (FS-6) was purchased from Merck Millipore (Watford, UK), whilst the quenched fluorescent peptide 2-Abz-SKGRSLIG-Y(NO_2_) was synthesized by GL Biochem (Shanghai, China).

### Cartilage explant culture

Explants were taken from macroscopically normal areas of cartilage from OA patients, undergoing total knee replacement at the Freeman Hospital, Newcastle. All tissue was obtained following informed consent from patients, and processing and handling carried out in accordance with University guidelines with appropriate ethical approval from NHS Health Authority NRES Committee North East – Newcastle and North Tyneside 1 (REC14/NE/1212). Cartilage was prepared and cultured as described previously^6^, and stimulated with recombinant TTSP for either 3 or 7 days. For bovine cartilage explants, nasal septum (obtained from a local abattoir) was prepared as previously described^20^. Two discs were plated in each well of a 48-well plate and incubated in serum-free medium overnight. Cartilage was stimulated with IL-1 + OSM (1 ng/ml and 10 ng/ml, respectively) in the presence or absence of matriptase or hepsin (100 nM). Cartilage was re-stimulated at day 7 and the experiment terminated following complete resorption of ≥1 of the wells (typically day 10–12). Media were removed and stored at −20 °C prior to analysis. The remaining cartilage was digested with papain^20^. To analyse cartilage destruction, hydroxyproline levels were used as an estimate for collagen release into conditioned medium, whilst aggrecan release was measured by assessing levels of glycosaminoglycan (GAG) by dimethyl methylene blue (DMMB) assay (see^20^ and the references therein). Collagenolytic activity present in culture media from cartilage explants was determined using a diffuse fibril assay with ^3^H acetylated collagen^45^. Gelatin zymography was performed as previously described^25^. For cytokine experiments, bovine articular explants were stimulated for a total of 96 hours with IL-1α (5 ng/ml) and oncostatin-M (10 ng/ml). Explants were snap frozen and homogenised in TRIzol® (Invitrogen, Paisley, UK) in liquid nitrogen using a dismembrator (Sartorious, Epsom, UK), RNA extracted and complementary DNA synthesised using Superscript III reverse transcriptase according to the manufacturer’s instructions (Invitrogen). Real-time PCR was performed using a MxPro3000 QPCR system (Agilent Technologies, Stockport, UK) using ReadyMix™ Taq PCR reaction mix in conjunction with a Taqman® gene expression assay (Hpn: Bt03260765_m1; ThermoFisher Scientific (Loughborough, UK)). *18 S* was used as a reference gene as previously described^46^.

### ProMMP activation and SDS-PAGE

Recombinant hepsin and matriptase were incubated with proMMP-1, proMMP-8, proMMP-9 and proMMP-13 at a 1:5 enzyme to MMP ratio whilst proMMP-3 was at a 1:10 ratio. Each proMMP was activated with 4-aminophenylmercuric acetate (APMA; 0.67 mM) as a positive control^24^. To demonstrate the requirement for serine proteinase activity, TTSPs were also pre-treated with the irreversible serine proteinase inhibitor di-isopropyl fluorophosphate (DFP; 2 mM). Each preparation was adjusted to a final volume of 20 µl in 100 mM Tris pH 8.0, 150 mM NaCl, 10 mM CaCl_2_, 0.01% (w/v) Brij 35 and incubated for 4 hours at 37 °C. Resultant MMP activity was assessed by the MMP-specific quenched fluorescent peptide, FS-6. Briefly, samples were incubated in duplicate at 37 °C in 100 mM Tris pH 7.5, 100 mM NaCl, 10 mM CaCl_2_, 0.01% (w/v) Brij 35, 0.1% (v/v) polyethylene glycol 6000 prior to FS-6 addition (50 µM final concentration) in 100 µl final volume. The reaction was allowed to proceed at 37 °C until approximately 10% substrate hydrolysis was reached, before being stopped by the addition of 50 µl 2 M sodium acetate. Fluorescence was monitored at 325 nm λ_excitation_ and 405 nm λ_emission_ in a Perkin Elmer LS50B plate reader. For time-course experiments, hepsin was incubated with proMMP-1 and proMMP-3 in a 1:5 hepsin:proMMP ratio, and aliquots taken at various time points from 30 minutes to 24 hours.

Proteins were resolved by sodium dodecyl sulphate polyacrylamide gel electrophoresis (SDS-PAGE) using a Mini-Protean tetra-cell apparatus (BioRad, Herts, UK) with separate resolving (10% (w/v) acrylamide/bisacrylamide (37.5:1), 375 mM Tris HCl, pH 8.8, and 0.1% (w/v) SDS) and stacking (4.5% (w/v) acrylamide/bisacrylamide (37.5:1), 125 mM Tris HCl, pH 6.8, 0.1% (w/v) SDS) gels. Samples, including molecular mass markers (5 μl: PageRuler Prestained, Thermofisher) were reduced and boiled in 5x SDS-PAGE loading buffer (625 mM Tris HCl pH 6.8, 10% (w/v) SDS, 40% (v/v) glycerol, 0.5% (w/v) bromophenol blue, 5% (v/v) β-mercaptoethanol) for 5 minutes prior to electrophoresis in 1 mm thick gels using 25 mM Tris HCl, 20 mM glycine, 1% (w/v) SDS running buffer. Following separation, gels were stained with silver according to the manufacturer’s instructions (Plus One silver staining kit, GE healthcare, Buckinghamshire, UK).

### Aggrecan digestion

Deglycosylated aggrecan was extracted from bovine nasal cartilage as described previously^47^. Recombinant hepsin or matriptase (both 10 nM) were incubated with 400 nM aggrecan in 100 μl final volume made up with 100 mM Tris pH 8.5, 150 mM NaCl, 0.01% (w/v) Brij 35) and incubated for 16 hours. Products were reduced, boiled, separated by SDS-PAGE (as described above), semi-dry blotted onto PVDF and western blotting carried out using an anti-G1 antibody. For proteolysis of recombinant G1-IGD-G2 (R&D Systems), proteinases were incubated at a 1:20 enzyme to substrate ratio (7.8 nM and 156 nM, respectively) in a final volume of 120 μl. Cleavage products were separated by SDS-PAGE and first stained with silver or immunoblotted using aggrecan neo-epitope antibodies recognizing MMP- or aggrecanase-specific cleavages. In subsequent experiments, digestion products were transferred to PVDF and either stained for 2 minutes with Coomassie Brilliant Blue R250, or immunoblotted with an anti-G1 antibody to assist identification of the specific band of interest which was excised and N-terminal sequenced (Cambridge Peptides, Birmingham, UK).

### Synovial Tissue and Immunohistochemistry

Human synovia were generously provided by Prof Ian Clark (University of East Anglia, Norwich, UK) from patients undergoing total hip replacement surgery at the Norwich and Norfolk Hospital, Norwich, UK for OA (6 M/6 F; mean age = 74.3 yrs (±3.6 SD), range 68–78 yrs) or NOF fracture (2 M/10 F; mean age = 76 yrs (±10 SD), range 52–89 yrs). Human knee synovia were obtained from OA patients undergoing total knee replacement surgery (3 M/8 F; mean age = 65.6 yrs (±8.4 SD), range 54–86 yrs), at the Freeman Hospital, Newcastle, UK. All tissue was obtained following informed consent from patients, and processing carried out in accordance with University guidelines and with ethical approval from either Norfolk Research Ethics Committee (REC05/Q010/196) or NHS Health Authority NRES Committee North East – Newcastle and North Tyneside 1 (REC14/NE/1212). RNA was isolated from hip synovia and subjected to TaqMan low density array PCR as previously described^5,48^. Knee synovial tissue was fixed overnight in 10% formalin and paraffin embedded as described^6^. Sections were generated at 4 μm intervals and stained with haemotoxylin and eosin as described previously^19^. Synovitis was scored according to a previously described system^49^. For immunohistochemistry, sections were de-waxed in xylene followed by decreasing alcohol gradients and antigen retrieval performed by immersion in hot 10 mM sodium citrate, pH 6.0, for 6 minutes. Endogenous peroxidase activity was quenched in 0.3% (v/v) H_2_O_2_ for 30 minutes. A biotin block was performed according to the manufacturer’s instructions (Vector Laboratories, CA, USA), followed by blocking in 5% horse serum. Hepsin antibody at 1:100 dilution (Cayman Chemicals, Michigan, USA) was applied for 30 minutes. After washing, detection was performed using an ABC peroxidase staining kit (Thermofisher Scientific, Loughborough, UK) according to the manufacturer’s instructions, followed by incubation with 3, 3′-diaminobenzidine tetra-hydrochloride (DAKO, Ely, UK) for 5 minutes. Sections were washed in tap water, counterstained with Mayer’s haemotoxylin and slides mounted. Images were taken using a Leica Biosystems DM4000B light microscope.

### PAR2 assays

All mice were C57BL/6 J background, and housed in standard cages. All procedures were approved and conducted in accordance with UK Home Office legislation (project licence number 70/8790) and institutional regulations. Food and water was available *ad libitum* and all animals were kept in a thermoneutral environment. In *vivo* PAR2 activation was assessed by consecutive laser Doppler imaging (Moor Instruments, Axminster, UK) of synovial perfusion following administration of hepsin (5 µg) to PAR2^+/+^ and PAR2^−/−^ mice, as described previously^5^. For cell experiments, full-length PAR2 was amplified from a commercially available shuttle vector (R&D Systems, Abingdon, UK) and cloned into a pcDNA3.1 vector using an Infusion-HD kit (Clontech, CA, USA) according to the manufacturer’s protocol. 5′-TACCGAGCTCGGATCGCCACCATGGACTACAAGGATGACGATGACAAGCGAAGTCCTAGTGCT-3′ and 5′-AAACGGGCCCTCTAGTTAATAGGAGGTCTTAACAG-3′ were used as forward and reverse primers, respectively, which provided an N-terminal FLAG tag and kozak sequence. SW1353 chondrosarcoma cells were cultured as described previously^50^. For transfection, 2.75 µg plasmid DNA with 8.25 µl Fugene-HD transfection reagent (Promega, Southampton, UK) were added to cells for 48 hours in antibiotic-free medium followed by selection in medium containing G418 disulphate (neomycin analogue, 400 µg/ml). Cells were incubated with matriptase or hepsin (0–100 nM) for 3 hours. RNA was stabilised in Cells-to-cDNA lysis buffer and converted to cDNA by Moloney-Murine Leukemia Virus reverse transcriptase as directed (Invitrogen). *IL-8* (*CXCL8*) expression was monitored by TaqMan real-time PCR (forward primer: 5′-AGACAGCAGAGCACACAAGC-3′; reverse primer: 5′AGGAAGGCTGCCGAGAG-3′; Probe: #72 from Universal Probe Library (Roche, Welwyn Garden City, UK), and normalised to *18 S* ribosomal RNA levels (primers and probe as described in^51^). The kinetics of PAR2 cleavage by hepsin or matriptase (both 1 nM) were assessed in pre-warmed 100 mM Tris pH 7.5, 150 mM NaCl, 500 µg/ml bovine serum albumin, 0.01% (w/v) Brij 35 before addition of 2-Abz-SKGRSLIG-Y(NO_2_) at a range of concentrations (up to 200 µM). Progress curves were monitored over 30 minutes at 37 °C, and slopes from the linear portions of each curve plotted against substrate concentration and kinetic constants generated by non-linear regression using GraphPad Prism 7.0 software.

### Molecular Modelling

All operations were performed within the Schrödinger Molecular Modelling Suite version 2017-1 (NY, USA). Crystal structures of matriptase and hepsin were downloaded from the Protein Data Bank (3PF8 and 1ZG8, respectively) and aligned using the backbone atoms of chain A. The amino acid sequence RCTKSI of the bound peptide (chain I) was extracted from 3PF8 and mutated to the PAR2 cleavage sequence SKGRSL. The termini of the resulting PAR2 peptide were amide capped and restrained minimization performed using Impact. Glide SP was used to obtain a rough positioning of the peptide in the binding sites of matriptase and hepsin (flexible peptide, rigid receptor), followed by refinement using Prime MM-GBSA, allowing simultaneous sampling of peptide and receptor, with full flexibility for residues within 6 Å of the peptide.

### Statistical analysis

Statistical differences between sample groups were assessed using ANOVA with a post-hoc Bonferroni’s multiple comparison test. For comparisons between only two samples, a two-tailed Student’s t-test was performed. A Pearson correlation co-efficient calculation was performed for synovial gene expression data. All statistical analyses were performed using Graphpad Prism 7.0 software where ***p < 0.001; **p < 0.01; *p < 0.05.

### Equipment and Settings

All gel and blot images were taken using Syngene using GeneSnap software. Western blots were detected after 5 minutes in enhanced chemiluminescent (ECL) substrate. Images were captured under ‘no light’ conditions with 5 × 30 seconds exposure time. Upper white images were taken to determine the position of the ladder, and composite images generated using GeneSnap. Images of silver stain gels were taken using the ‘transilluminator’ setting, using a visible light converter screen to produce an evenly illuminated area of white light. Any changes to brightness or contrast were applied uniformly across the whole gel or blot.

### Data Availability

The datasets generated/analysed during the current study are available from the corresponding author on reasonable request.

## Electronic supplementary material

Supplementary Information

## References

[1] Rowan AD, Litherland GJ, Hui W, Milner JM (2008). Metalloproteases as potential therapeutic targets in arthritis treatment. Expert Opin Ther Targets.

[2] Jubb RW, Fell HB (1980). The breakdown of collagen by chondrocytes. J Pathol.

[3] Hollander AP (1995). Damage to type II collagen in aging and osteoarthritis starts at the articular surface, originates around chondrocytes, and extends into the cartilage with progressive degeneration. J Clin Invest.

[4] Milner JM, Patel A, Rowan AD (2008). Emerging roles of serine proteinases in tissue turnover in arthritis. Arthritis Rheum.

[5] Milner JM (2010). Matriptase is a novel initiator of cartilage matrix degradation in osteoarthritis. Arthritis Rheum.

[6] Wilkinson, D. J. *et al*. Matriptase induces metalloproteinase-dependent aggrecanolysis *in vitro* and *in vivo*: multiple mechanisms promote cartilage damage in osteoarthritis. *Arthritis Rheumatol*, 10.1002/art.40133 (2017). 10.1002/art.40133 PMC5599990 28464560

[7] Ferrell WR (2003). Essential role for proteinase-activated receptor-2 in arthritis. J Clin Invest.

[8] Ferrell WR, Kelso EB, Lockhart JC, Plevin R, McInnes IB (2010). Protease-activated receptor 2: a novel pathogenic pathway in a murine model of osteoarthritis. Ann Rheum Dis.

[9] Huesa C (2016). Proteinase-activated receptor 2 modulates OA-related pain, cartilage and bone pathology. Ann Rheum Dis.

[10] Kelso EB (2007). Expression and proinflammatory role of proteinase-activated receptor 2 in rheumatoid synovium: *ex vivo* studies using a novel proteinase-activated receptor 2 antagonist. Arthritis Rheum.

[11] Xiang Y (2006). Expression of proteinase-activated receptors (PAR)-2 in articular chondrocytes is modulated by IL-1beta, TNF-alpha and TGF-beta. Osteoarthritis Cartilage.

[12] Beliveau F, Desilets A, Leduc R (2009). Probing the substrate specificities of matriptase, matriptase-2, hepsin and DESC1 with internally quenched fluorescent peptides. FEBS J.

[13] Herter S (2005). Hepatocyte growth factor is a preferred *in vitro* substrate for human hepsin, a membrane-anchored serine protease implicated in prostate and ovarian cancers. Biochem J.

[14] Moran P (2006). Pro-urokinase-type plasminogen activator is a substrate for hepsin. J Biol Chem.

[15] Uhland K (2006). Matriptase and its putative role in cancer. Cell Mol Life Sci.

[16] Antalis TM, Bugge TH, Wu Q (2011). Membrane-anchored serine proteases in health and disease. Prog Mol Biol Transl Sci.

[17] Camerer E (2010). Local protease signaling contributes to neural tube closure in the mouse embryo. Dev Cell.

[18] Tanabe LM, List K (2017). The role of type II transmembrane serine protease-mediated signaling in cancer. FEBS J.

[19] Barksby HE (2006). Matrix metalloproteinase 10 promotion of collagenolysis via procollagenase activation: implications for cartilage degradation in arthritis. Arthritis Rheum.

[20] Cawston, T. E. *et al*. The role of oncostatin M in animal and human connective tissue collagen turnover and its localization within the rheumatoid joint. *Arthritis Rheum* **41**, 1760–1771, 10.1002/1529-0131 (1998). 10.1002/1529-0131(199810)41:10<1760::AID-ART8>3.0.CO;2-M 9778217

[21] Koshy PJ (2002). The modulation of matrix metalloproteinase and ADAM gene expression in human chondrocytes by interleukin-1 and oncostatin M: a time-course study using real-time quantitative reverse transcription-polymerase chain reaction. Arthritis Rheum.

[22] Mathiessen A, Conaghan PG (2017). Synovitis in osteoarthritis: current understanding with therapeutic implications. Arthritis Res Ther.

[23] Rawlings ND, Barrett AJ, Finn R (2016). Twenty years of the MEROPS database of proteolytic enzymes, their substrates and inhibitors. Nucleic Acids Res.

[24] Milner, J. M., Elliott, S. F. & Cawston, T. E. Activation of procollagenases is a key control point in cartilage collagen degradation: interaction of serine and metalloproteinase pathways. *Arthritis Rheum* **44**, 2084–2096, 10.1002/1529-0131 (2001). 10.1002/1529-0131(200109)44:9<2084::AID-ART359>3.0.CO;2-R 11592371

[25] Milner JM, Rowan AD, Elliott SF, Cawston TE (2003). Inhibition of furin-like enzymes blocks interleukin-1alpha/oncostatin M-stimulated cartilage degradation. Arthritis Rheum.

[26] Milner JM, Rowan AD, Cawston TE, Young DA (2006). Metalloproteinase and inhibitor expression profiling of resorbing cartilage reveals pro-collagenase activation as a critical step for collagenolysis. Arthritis Res Ther.

[27] Klezovitch O (2004). Hepsin promotes prostate cancer progression and metastasis. Cancer Cell.

[28] Wu Q, Parry G (2007). Hepsin and prostate cancer. Front Biosci.

[29] Mittal, R. *et al*. Intricate Functions of Matrix Metalloproteinases in Physiological and Pathological Conditions. *J Cell Physiol*, 10.1002/jcp.25430 (2016). 10.1002/jcp.25430 27187048

[30] Tervonen TA (2016). Deregulated hepsin protease activity confers oncogenicity by concomitantly augmenting HGF/MET signalling and disrupting epithelial cohesion. Oncogene.

[31] Loeser RF (2013). Disease progression and phasic changes in gene expression in a mouse model of osteoarthritis. PLoS One.

[32] Malfait AM (2016). Osteoarthritis year in review 2015: biology. Osteoarthritis Cartilage.

[33] Robinson WH (2016). Low-grade inflammation as a key mediator of the pathogenesis of osteoarthritis. Nat Rev Rheumatol.

[34] Barksby HE (2006). Interleukin-1 in combination with oncostatin M up-regulates multiple genes in chondrocytes: implications for cartilage destruction and repair. Arthritis Rheum.

[35] Knauper V, Lopez-Otin C, Smith B, Knight G, Murphy G (1996). Biochemical characterization of human collagenase-3. J Biol Chem.

[36] Murphy G, Cockett MI, Stephens PE, Smith BJ, Docherty AJ (1987). Stromelysin is an activator of procollagenase. A study with natural and recombinant enzymes. Biochem J.

[37] He CS (1989). Tissue cooperation in a proteolytic cascade activating human interstitial collagenase. Proc Natl Acad Sci USA.

[38] Qiu D, Owen K, Gray K, Bass R, Ellis V (2007). Roles and regulation of membrane-associated serine proteases. Biochem Soc Trans.

[39] Barre O (2014). Cleavage specificity analysis of six type II transmembrane serine proteases (TTSPs) using PICS with proteome-derived peptide libraries. PLoS One.

[40] Lees S (2015). Bioactivity in an Aggrecan 32-mer Fragment Is Mediated via Toll-likeReceptor 2. Arthritis Rheumatol.

[41] Rousselle P, Beck K (2013). Laminin 332 processing impacts cellular behavior. Cell Adh Migr.

[42] Xuan JA (2006). Antibodies neutralizing hepsin protease activity do not impact cell growth but inhibit invasion of prostate and ovarian tumor cells in culture. Cancer Res.

[43] Malfait AM, Little CB (2015). On the predictive utility of animal models of osteoarthritis. Arthritis Res Ther.

[44] Ganesan R (2012). An allosteric anti-hepsin antibody derived from a constrained phage display library. Protein Eng Des Sel.

[45] Koshy PJ, Rowan AD, Life PF, Cawston TE (1999). 96-Well plate assays for measuring collagenase activity using (3)H-acetylated collagen. Anal Biochem.

[46] Al-Sabah A, Stadnik P, Gilbert SJ, Duance VC, Blain EJ (2016). Importance of reference gene selection for articular cartilage mechanobiology studies. Osteoarthritis Cartilage.

[47] Hui W (2005). Oncostatin M in combination with tumour necrosis factor {alpha} induces a chondrocyte membrane associated aggrecanase that is distinct from ADAMTS aggrecanase-1 or -2. Ann Rheum Dis.

[48] Swingler TE (2009). Degradome expression profiling in human articular cartilage. Arthritis Res Ther.

[49] Krenn V (2002). Grading of chronic synovitis–a histopathological grading system for molecular and diagnostic pathology. Pathol Res Pract.

[50] Radwan M (2013). Matrix metalloproteinase 13 expression in response to double-stranded RNA in human chondrocytes. Arthritis Rheum.

[51] Litherland GJ (2008). Synergistic collagenase expression and cartilage collagenolysis are phosphatidylinositol 3-kinase/Akt signaling-dependent. J Biol Chem.

[52] Tripathi M (2008). Laminin-332 is a substrate for hepsin, a protease associated with prostate cancer progression. J Biol Chem.

[53] McMahon BJ, Kwaan HC (2015). Components of the Plasminogen-Plasmin System as Biologic Markers for Cancer. Adv Exp Med Biol.

[54] Yoshida N (2007). Interleukin-8 production via protease-activated receptor 2 in human esophageal epithelial cells. Int J Mol Med.

[55] Nagase H, Enghild JJ, Suzuki K, Salvesen G (1990). Stepwise activation mechanisms of the precursor of matrix metalloproteinase 3 (stromelysin) by proteinases and (4-aminophenyl)mercuric acetate. Biochemistry.

