# Supplementary material for: The serine proteinase hepsin is an activator of pro-matrix metalloproteinases: molecular mechanisms and implications for extracellular matrix turnover
Source: Sci Rep. 2017 Dec 1;7:16693. doi: 10.1038/s41598-017-17028-3 (PMC5711915; doi:10.1038/s41598-017-17028-3)
Supplement: Supplementary file 1 — Supplementary Information [file 41598_2017_17028_MOESM1_ESM.pdf]

<u>**Supplementary Information**</u>

The serine proteinase hepsin is an activator of pro-matrix metalloproteinases: molecular mechanisms and implications for extracellular matrix turnover

David J. Wilkinson, Antoine Desilets, Hua Lin, Sarah Charlton, Maria del Carmen Arques, Adrian Falconer, Craig Bullock, Yu-Chen Hsu, Kristian Birchall, Alastair Hawkins, Paul Thompson, William R. Ferrell, John Lockhart, Robin Plevin, Yadan Zhang, Emma Blain, Shu-Wha Lin, Richard Leduc, Jennifer M. Milner, and Andrew D. Rowan

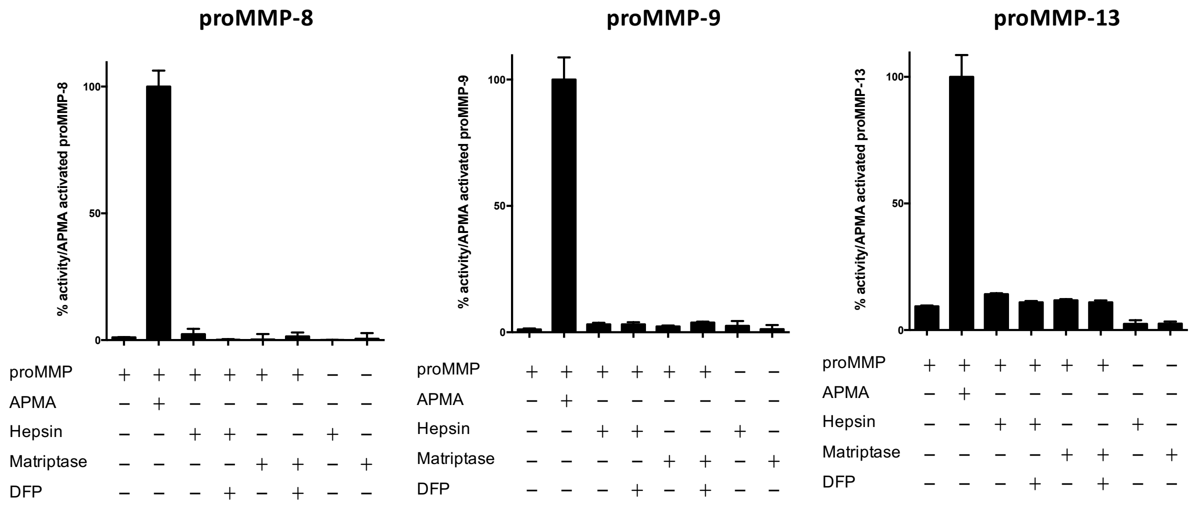

Supplementary Figure 1. Hepsin does not activate proMMP-8, proMMP-9 and proMMP-13. For activity assays, proMMP-8, proMMP-9 or proMMP-13 were incubated with hepsin or matriptase for 4 h at 37°C. Enzyme to substrate ratios were 1:5 with final concentrations of proMMP-8, proMMP-9 and proMMP-13 being 76 nM, 276 nM, 111 nM respectively. Each preparation was made up to a final volume of 20 μl in buffer (100 mM Tris pH 8.0, 150 mM NaCl, 10 mM $CaCl_2$, 0.01% (w/v) Brij 35) and incubated for 4 hours at 37°C. MMP activity was then assessed with the MMP-specific substrate FS-6. Briefly, 7 μl of incubation products were added to a white-walled plate at 37°C in a total volume of 80 μl of buffer (100 mM Tris HCl pH 7.5, 100 mM NaCl, 10 mM $CaCl_2$, 0.01% (w/v) Brij 35, 0.1% (v/v) polyethylene glycol 6000). FS-6 at a final concentration of 50 μM was then added to a 100 μl total volume. The reaction was allowed to proceed at 37°C until approximately 10% substrate hydrolysis was reached, before being stopped by the addition of 50 μl 2 M sodium acetate. Fluorescence was monitored at 325nm $\lambda_{excitation}$ and 405nm $\lambda_{emission}$ in a Perkin Elmer LS50B plate reader. ProMMP alone or in the presence of APMA were used as negative and positive controls, respectively. Hepsin and matriptase without MMP did not cleave the substrate significantly. Hepsin and matriptase were also pre-treated with the irreversible serine proteinase inhibitor di-isopropyl fluorophosphate (DFP), to demonstrate the involvement of serine proteinase activity in MMP activation. Data are presented as a percentage of APMA-activated proMMP (mean ± SD) following subtraction of the blank measurement.

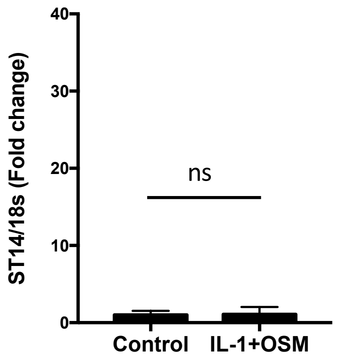

Supplementary Figure 2. Matriptase expression is not induced in bovine articular cartilage following stimulation with pro-inflammatory cytokines IL-1 +OSM. RNA extraction and reverse transcription were conducted as detailed for hepsin in the Methods section. Real-time PCR was performed using an assay-on-demand kit (ThermoFisher; Bt03249536_m1) and normalised to 18S rRNA. Statistical analysis performed by two-tailed t-test where ns = not significant.

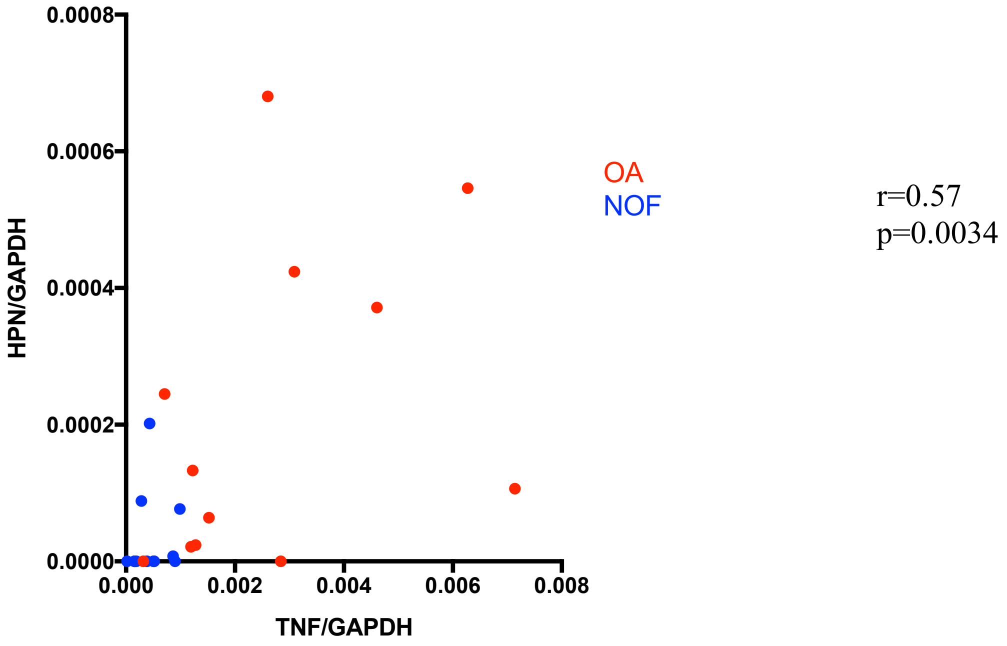

Supplementary Figure 3. Hepsin expression correlates with TNF expression. Expression levels of hepsin and TNF in human hip synovia from OA patients and control neck-of-femur (NOF) fracture were determined by TaqMan Low Density Array. Expression levels for each sample were correlated and a Pearson's correlation coefficient (r) was determined using GraphPad Prism 7.0 software.

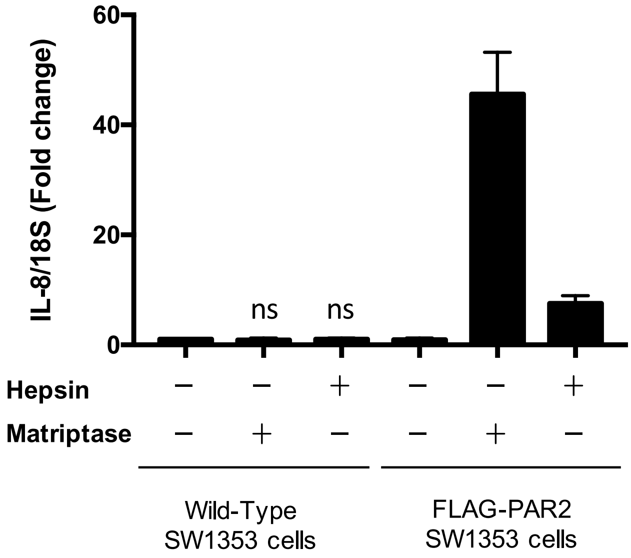

Supplementary Figure 4. Cellular response to protease is dependent on PAR2 overexpression. Hepsin or matriptase (50 nM) was added to wild-type cells or cells with a stable overexpression of FLAG-PAR2. Cells were lysed, RNA reverse transcribed and real-time PCR performed as described in the Methods section. Neither hepsin or matriptase induced significant expression of the PAR2 responsive gene, IL-8, in wild-type SW1353 cells. Data are represented as fold change versus unstimulated control ± standard deviation. Statistical analysis performed using a one-way ANOVA with Bonferroni post-hoc test where ns = not significant. For clarity only selected comparisons are shown.

Supplementary Figure 5. Hepsin-deficient mice or hepsin inhibition in wild-type mice are not protected against cartilage damage in the DMM model. A. OA was induced in C57BL/6J, *Hpn*$^{+/+}$ mice following DMM surgery (n = 16) whilst some animals only received sham surgery (n = 6). In parallel, some animals received 0.9% saline alone (n = 5) or hepsin inhibitory antibody Ab 25 in 0.9% saline at 10 µg/day (n = 6), administered via subcutaneous osmotic mini-pumps. Similarly, *Hpn*$^{-/-}$ (C57BL/6J background) were subjected to DMM (n = 24) or sham surgery (n = 6), and all animals sacrificed at 8 weeks post-surgery. Consecutive knee joint sections (4 µm) were stained with hematoxylin-Safranin O-fast green. Images are representative of animals in each treatment group. No significant differences in cartilage damage scores following were observed in any of the different genotypes or treatment groups.

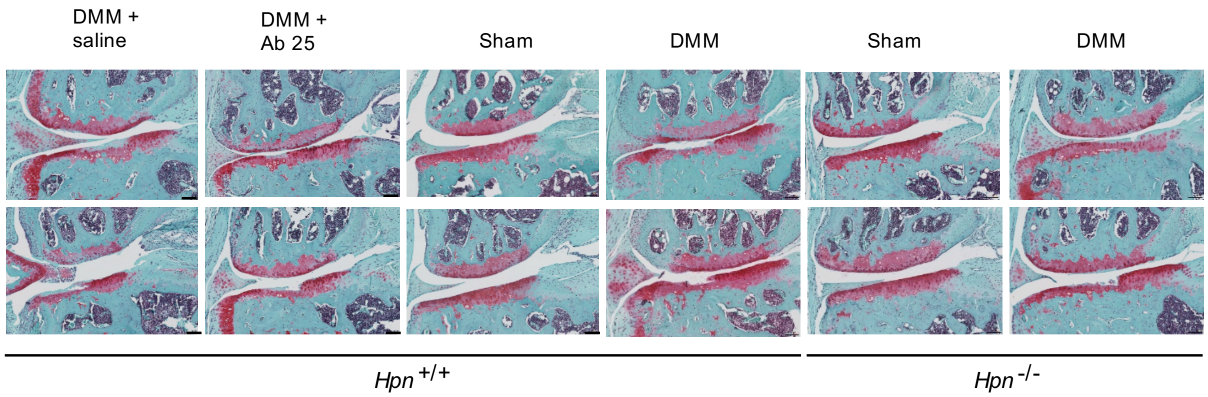

Additional Methods for Supplementary Figure 5. All mice were C57BL/6J background, and housed in standard cages. *Hpn*$^{+/-}$ mice[1] were bred to generate homozygous, hepsin-deficient animals prior to use in experiments. Food and water were available *ad libitum*. All procedures were performed in accordance with current UK Home Office legislation and institutional regulations. Surgical destabilisation of the medial meniscus (DMM) surgery was performed on 10 week-old male mice to induce experimental OA[2]. The Ab25 hepsin neutralising antibody[3] was delivered via an intraperitoneal osmotic pump (Alzet 1004; Charles River, Margate, UK) of 100 µl capacity delivering 10 µg of antibody (in 0.9% saline)/day at a rate of 0.11 µl/h for 4 weeks, after which time fresh osmotic pumps were inserted as before with identical contents for a further 4 weeks. Mice were then euthanized and knee joints harvested for histological examination. Cartilage damage was graded (by two blinded, independent observers) according to a system used previously[4].

1 Yu, I. S. *et al.* Mice deficient in hepsin, a serine protease, exhibit normal embryogenesis and unchanged hepatocyte regeneration ability. *Thromb Haemost* **84**, 865-870 (2000).

2 Glasson, S. S., Blanchet, T. J. & Morris, E. A. The surgical destabilization of the medial meniscus (DMM) model of osteoarthritis in the 129/SvEv mouse. *Osteoarthritis Cartilage* **15**, 1061-1069, doi:10.1016/j.joca.2007.03.006 (2007).

3 Ganesan, R. *et al.* An allosteric anti-hepsin antibody derived from a constrained phage display library. *Protein Eng Des Sel* **25**, 127-133, doi:10.1093/protein/gzr067 (2012).

4 Glasson, S. S., Chambers, M. G., Van Den Berg, W. B. & Little, C. B. The OARSI histopathology initiative - recommendations for histological assessments of osteoarthritis in the mouse. *Osteoarthritis Cartilage* **18 Suppl 3**, S17-23, doi:10.1016/j.joca.2010.05.025 (2010).

Full length Gels and Blots

Figure 2B

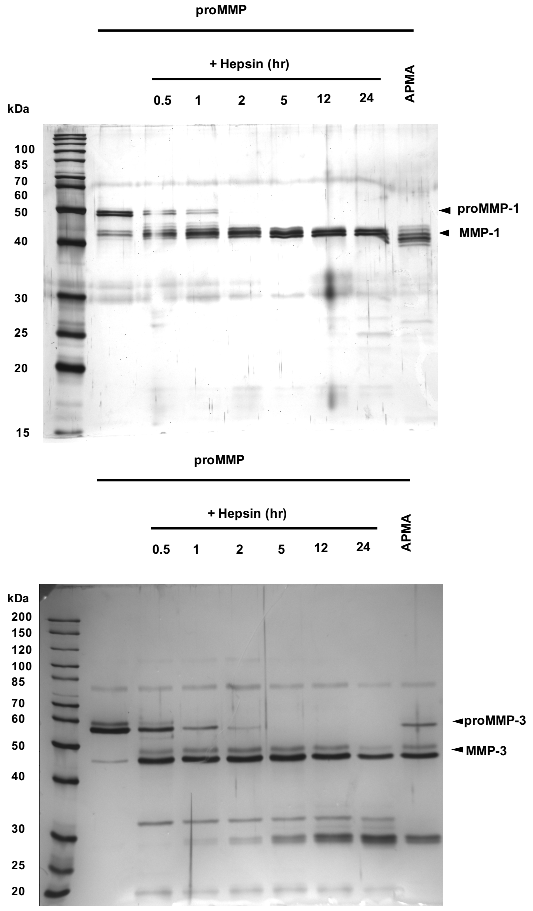

Figure 2C

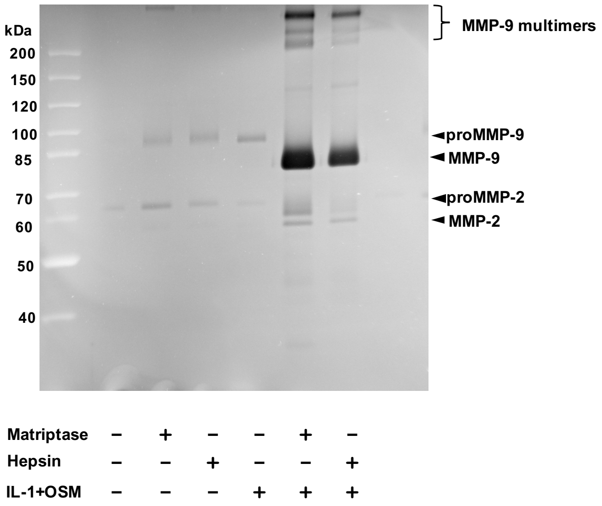

Figure 3A

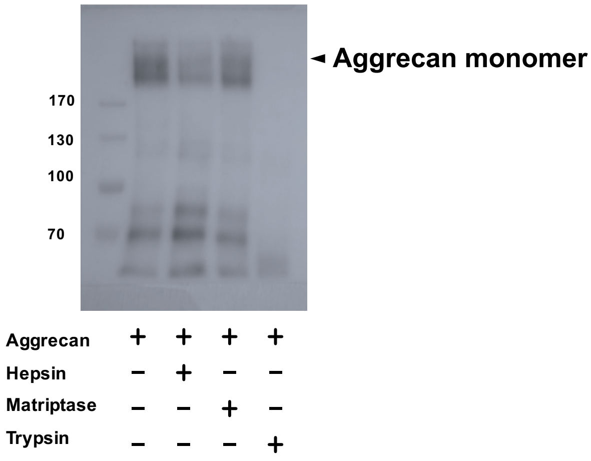
Aggrecan monomer
170
130
100
70
Aggrecan + + + +
Hepsin − + − −
Matriptase − − + −
Trypsin − − − +
